# Impact of histological response after neoadjuvant therapy on podocalyxin as a prognostic marker in pancreatic cancer

**DOI:** 10.1038/s41598-021-89134-2

**Published:** 2021-05-10

**Authors:** Annika Eurola, Ari Ristimäki, Harri Mustonen, Anna-Maria Nurmi, Jaana Hagström, Caj Haglund, Hanna Seppänen

**Affiliations:** 1 Department of Surgery, Translational Cancer Medicine Research Program, Faculty of Medicine, University of Helsinki and Helsinki University Hospital, Haartmaninkatu 4, PO Box 340, 00029 HUS Helsinki, Finland; 2 Department of Pathology, HUSLAB, HUS Diagnostic Center, University of Helsinki and Helsinki University Hospital, Helsinki, Finland; 3 Applied Tumor Genomics (ATG), Research Programs Unit, Faculty of Medicine, University of Helsinki, Helsinki, Finland; 4 Department of Oral Pathology and Radiology, University of Turku, Turku, Finland

**Keywords:** Pancreatic cancer, Prognostic markers, Tumour biomarkers

## Abstract

Podocalyxin overexpression associates with poor survival in pancreatic cancer (PDAC). We investigated whether podocalyxin expression correlates with treatment response or survival in neoadjuvant-treated PDAC. Through immunohistochemistry, we evaluated podocalyxin expression in 88 neoadjuvant and 143 upfront surgery patients using two antibodies. We developed a six-tier grading scheme for neoadjuvant responses evaluating the remaining tumor cells in surgical specimens. Strong podocalyxin immunopositivity associated with poor survival in the patients responding poorly to the neoadjuvant treatment (HR 4.16, 95% CI 1.56–11.01, p = 0.004), although neoadjuvant patients exhibited generally low podocalyxin expression (p = 0.017). Strong podocalyxin expression associated with perineural invasion (p = 0.003) and lack of radiation (p = 0.036). Two patients exhibited a complete neoadjuvant response, while a strong neoadjuvant response (≤ 5% of residual tumor cells) significantly associated with lower stage, pT-class and grade, less spread to the regional lymph nodes, less perineural invasion, and podocalyxin negativity (p < 0.05, respectively). A strong response predicted better survival (HR 0.28, 95% CI 0.09–0.94, p = 0.039). In conclusion, strong podocalyxin expression associates with poor survival among poorly responding neoadjuvant patients. A good response associates with podocalyxin negativity. A strong response associates with better outcome.

## Introduction

Pancreatic cancer has now become the third most common cause of cancer-related death. In recent decades, the incidence of pancreatic cancer has increased, and survival rates remain poor and largely unchanged, with an overall five-year survival rate of 6% to 10%^1^. Under the most optimal situation, with localized, resectable disease, five-year survival can reach up to 37%^2–4^.

Many studies have shown that neoadjuvant therapy (NAT) is safe and effective for locally advanced and borderline-resectable disease^5–9^. Assessing the histological NAT effect in a post-pancreatectomy pancreatic ductal adenocarcinoma (PDAC) specimen is challenging, since some histological features of the treatment response, such as necrosis, fibrosis, and tumor cell atypia, overlap with features seen in untreated PDAC^10^. Furthermore, few schemes for evaluating the histopathological grading of the residual, viable tumor cells in the post-pancreatectomy specimen have emerged. Such schemes rely on the percentage of visible, severely degenerative, or viable residual cancer cells in the specimen^11–14^. Among these schemes, the Evans and the College of American Pathologists (CAP) grading systems appear to correlate with overall and disease-free survival (DFS)^15–17^. These systems have been criticized, however, for their lack of precision, clarity, simplicity, and clinical utility^18^. Recently, the Amsterdam International Consensus Meeting provided an overview listing statements regarding neoadjuvant response scoring in pancreatic cancer. Among other statements, response should specifically assess residual, rather than regressive, tumor cells and the defining criteria describing the residual tumor cells should be objective and repeatable rather than subjective^19^.

A sialomucin, podocalyxin is a transmembrane protein closely related to hematopoietic stem cell marker CD34^20^. It was first identified in the glomeruli, where it maintains the structure of podocytes and filtration slits through charge repulsion resulting from its negative charge^21–23^. Podocalyxin also appears in other normal tissues like the vascular endothelia^24^ and hematopoietic stem cells^25^. Podocalyxin expression has been reported in numerous malignancies including PDAC^26–31^, colorectal carcinoma^32–35^, hepatocellular carcinoma^36,37^, and gastric and esophageal cancers^38,39^. A high podocalyxin expression, in particular, associates with poor clinical outcomes in multiple cancers including colorectal cancer^32,33,35^, PDAC^28^, and gastric and esophageal cancers^38–41^.

The upregulation of podocalyxin appears necessary for the epithelial-mesenchymal transition, characterized by migratory and invasive behavior and involvement in metastatic events in cancer^41–43^. Furthermore, this upregulation appears to promote cancer cell proliferation, prevent apoptosis, promote tumorigenesis, and participate in cancer-cell renewal^40,41,44^. In addition, podocalyxin plays a role in prompting resistance to chemotherapeutic agents like cisplatin^45,46^, one of the drugs used to treat PDAC^8^. In PDAC, polyclonal (pAb) and monoclonal (mAb) antibodies were previously used to detect podocalyxin, and poor disease-specific survival (DSS) has been associated with both strong pAb and mAb staining as well as combined strong pAb–mAb staining^28^.

The role and underlying mechanisms of podocalyxin in neoadjuvant-treated cancer remain poorly understood. Podocalyxin is an independent factor predicting a poor prognosis in PDAC^28^, but has not been examined as an independent factor of prognosis in neoadjuvant-treated PDAC patients. The association between podocalyxin expression and earlier disease progression in PDAC remains unstudied, and whether podocalyxin works as a response marker following NAT is unknown. This study, therefore, aimed to clarify the relationship between podocalyxin and NAT in strong and poor NAT responders and the relationship between podocalyxin and disease progression in PDAC.

## Methods

We identified patients surgically treated for PDAC at Helsinki University Hospital between 2000 and 2016, finding 90 patients who received NAT and 144 propensity scored patients matched for age, sex, and time of surgery who underwent upfront surgery (US). Survival data and cause of death were collected from patient records, Statistics Finland, and the Finnish Population Registry. Patients who died of immediate postoperative complications were excluded (n=3).

### Tumor tissue microarray and immunohistochemistry

Formalin-fixed and paraffin-embedded post-pancreatectomy specimens were collected from hematoxylin-eosin stained samples and re-evaluated by experienced pathologists to confirm diagnosis and determine well-represented areas of PDAC for the tumor tissue microarray (TMA) preparation. Six 1.0-mm cores were drilled from each tumor with a semiautomatic tissue microarrayer (TMA Grand Master, TMA Control 3.0, 3D Histech, Hungary).

To identify and stain podocalyxin, two different antibodies that recognize two different amino acid residues were used. The monoclonal antibody (mAb) recognizes amino acid residues 189–192 of podocalyxin. It was created through the immunization of mice using undifferentiated human embryonic stem cells (hES), stem cell line SA167 (Cellartis, Gothenburg, Sweden, www.cellectis-bioresearch.com), and establishing mAb production against hES by conventional hybridoma technology. This process has been previously described in detail^35^. The polyclonal antibody (pAb) (HPA 2110, Atlas Antibodies, Stockholm, Sweden) recognizes amino acid residues 278–415. Both mAb and pAb have been validated^47,48^ and used in PDAC and podocalyxin studies before^26,28,35^.

TMA blocks were cut into 4-µm sections, deparaffinized in xylene and rehydrated through a gradually decreasing concentration of ethanol to distilled water. Slides were treated in a PreTreatment module (Lab Vision Corp., Fremont, and Ca, USA) in Tris-Hcl (pH 8.5) buffer for 20 min at 98°C for antigen retrieval. Slides were immunostained with pAb and mAb in an Autostainer 480 (Lab Vision Corp., Fremont, CA, USA) by the Dako REAL EnVision Detection system, Peroxidase/DAB+, rabbit/mouse (Dako, Glostrup, Denmark). Tissues were incubated with the mAb (1:500 = 2.4 mg/ml) and with the pAb (1:250 = 0.1 mg/ml) at room temperature for one hour. A sample of podocalyxin-positive colon cancer tissue served as the positive control.

**Figure 1:**
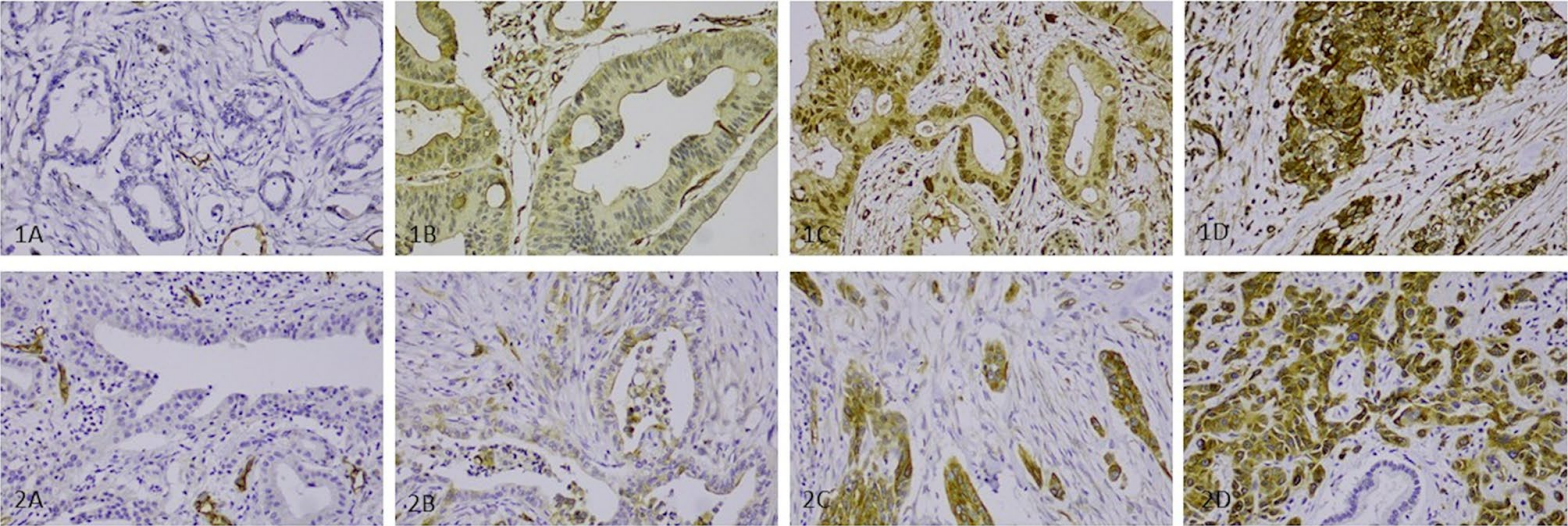
Immunohistochemical staining pattern of podocalyxin in neoadjuvant treated pancreatic ductal adenocarcinoma. Staining pattern of podocalyxin in neoadjuvant treated pancreatic ductal adenocarcinoma using monoclonal antibody, HES9 (**1A-1D**) and polyclonal antibody, HPA2110 (**2A-2D**). Representative images of podocalyxin expressions using HES9: (**1A**) negative expression with positivity in blood vessels, (**1B**) weak cytoplasmic positivity, (**1C**) moderate cytoplasmic positivity, and (**1D**) strong cytoplasmic positivity. Representative images of podocalyxin expression using HPA2110: (**2A**) negative expression with positivity in blood vessels, (**2B**) weak cytoplasmic positivity, (**2C**) moderate cytoplasmic positivity, and (**2D**) strong cytoplasmic positivity. Magnification is x200.

All TMA samples were evaluated by two independent, blinded investigators (AE and JH). The cytoplasmic staining intensity, in the PDAC cells, of podocalyxin stained with mAb and pAb was scored from 0 to 3: 0, negative; 1, weakly positive; 2, moderately positive; and 3, strongly positive (Fig. 1). The highest score across all six TMA samples was considered representative. In the case of differing scores, consensus was reached through re-evaluation.

### Grading the response to neoadjuvant therapy

All well-represented, diagnostic post-pancreatectomy samples from 88 NAT patients were collected. We designed a six-tier scheme to grade the response to NAT by evaluating the percentage of remaining, viable tumor cells: 0, no viable, residual tumor cells (RTCs) (0%); 1, only some found with large magnification of 200–400× (≤5% RTCs); 2, easily found with large magnification of 200–400× (6–10% RTCs); 3, easily found with small magnification of 20–40× (11–50% RTCs); 4, only little effect identified (51–90% RTCs); and 5, no effect identified (91–100% RTCs). Samples were evaluated by an experienced pathologist AR together with AE.

### Statistical analysis

For analytical purposes, a strong response to NAT was defined as a class 0 or 1 response, corresponding to ≤5% RTCs. In the survival analysis, non-responders were defined as a class 4 or 5 response corresponding to >50% RTCs, and responders as a class 0 to 3 response, ≤50% RTCs. Podocalyxin staining scores were combined and grouped as follows: 1, strong staining for both pAb and mAb (both scored 3); 2, either exhibiting strong staining (either scored 3); and 3, both staining weakly (both scored 0–2), corresponding to strong, moderate, and weak, respectively.

To compare categorical variables, we used the Fisher’s exact and linear-by-linear tests and relied on the Mann–Whitney U test for continuous variables. For the survival analysis, we used the Cox regression, Kaplan–Meier, and the Aalen–Johansen models. The log-rank and Gray’s tests were used to test for statistical significance. In the progression-free survival (PFS) analysis, progression was defined as the recurrence of cancer, deaths related to pancreatic cancer, and other deaths were used as competing events. We considered p < 0.05 as statistically significant. All statistical analyses were calculated using SPSS (version 25, IBM Corp., Armonk, TX, USA) and STATA/MP (version 16.1, StataCorp LLC, College Station, Texas, USA).

This study complies with the Declaration of Helsinki and was approved by the Surgical Ethics Committee of the Helsinki University Hospital (Dnro HUS 226/E6/06, extension TMK02 §66 17 April 2013). The National Supervisory Authority of Health and Welfare also granted permission (Valvira Dnro 10041/06.01.03.01/2012).

### Ethics approval

This study complies with the Declaration of Helsinki and was approved by the Surgical Ethics Committee of the Helsinki University Hospital (Dnro HUS 226/E6/06, extension TMK02 §66 17 April 2013). The National Supervisory Authority of Health and Welfare granted permission to use the tissue samples without requiring individual informed consent in this retrospective study (Valvira Dnro 10041/06.01.03.01/2012).

## Results

### Response to neoadjuvant therapy

Among 88 NAT patients, gemcitabine-cisplatin was the most used NAT (n = 43, 48.9%), either with (n = 26, 30%) or without radiation therapy. All NATs used are described in Supplementary Table 1.

**Figure 2:**
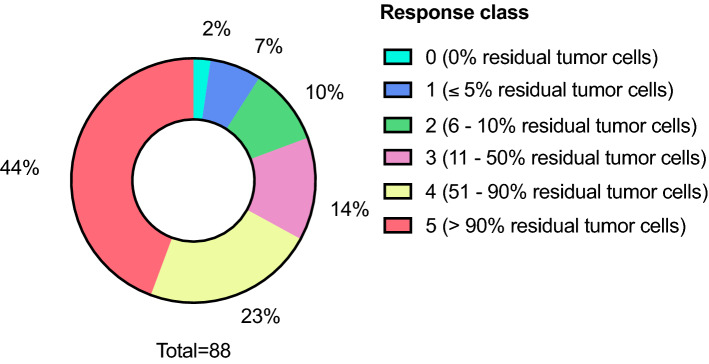
Frequencies of the different neoadjuvant responses and the percentage of viable residual tumor cells.

Among NAT patients, 2 (2.3%) had no viable residual tumor cells remaining and, thus, a complete response to NAT. A class 5 response with more than 90% of the RTCs remaining was identified in 39 (44.3%) patients, making it the most common response class. The distribution of all responses appears in Fig. 2.

A strong response to NAT was associated with neoadjuvant radiation (p = 0.046), lower stage (p = 0.015), lower pT-class (p = 0.005), less spread to the regional lymph nodes (p = 0.046), lower grade (p = 0.021), less perineural invasion (p = 0.009), and negative podocalyxin staining with both mAb (p = 0.023) and pAb (p = 0.008). Sex, age, vascular resection, and resection margin or perivascular invasion did not correlate with a strong response to NAT (Table 1).

**Table 1:** Neoadjuvant response associated with characteristics comparing strong responders (≤ 5% residual tumor cells) and poor responders (> 5% residual tumor cells). P-values representing the level of statistical significance are bolded.

n (%)	Strong response	Poor response	p value
	8 (9.1)	80 (90.9)	
**Sex**			
Female	4 (50.0)	45 (56.3)	
Male	4 (50.0)	35 (43.8)	1.000
**Age**			
< 65	5 (62.5)	37 (46.3)	
≥ 65	3 (37.5)	43 (53.8)	0.471
**Vascular resection**			
Yes	4 (50.0)	46 (57.7)	
No	4 (50.0)	34 (42.5)	0.722
**Radiation therapy**			
Yes	5 (62.5)	21 (26.3)	
No	3 (37.5)	59 (73.8)	**0.046**
**Stage**			
IA	3 (42.9)	7 (8.8)	
IB	3 (42.9)	22 (27.5)	
IIA	0 (0.0)	7 (8.8)	
IIB	0 (0.0)	30 (37.5)	
III	1 (12.5)	14 (17.5)	**0.015**
IV*			
Missing**	1		
**T**			
T1	3 (37.5)	17 (21.3)	
T2	3 (37.5)	44 (55.0)	
T3	0 (0.0)	17 (21.3)	
T4	1 (12.5)	2 (2.5)	**0.005**
Missing**	1		
**N**			
N0	7 (87.5)	37 (46.3)	
N1	1 (12.5)	31 (38.8)	
N2	0 (0.0)	12 (15.0)	**0.046**
**Grade**			
1	2 (25.0)	11 (13.8)	
2	3 (37.5)	52 (65.0)	
3	1 (12.5)	15 (18.8)	**0.021**
Missing***	4		
**Resection margin**			
R0	2 (25.0)	18 (23.1)	
R1	6 (75.0)	60 (76.9)	1.000
Missing***			
**Perineural invasion**			
Yes	2 (25.0)	59 (73.8)	
No	6 (75.0)	21 (26.3)	**0.009**
**Perivascular invasion**			
Yes	0 (0.0)	25 (31.3)	
No	8 (100.0)	55 (68.8)	0.099
**Negative pAb podocalyxin**			
Yes	5 (62.5)	13 (16.3)	
No	3 (37.5)	67 (83.8)	**0.008**
**Negative mAb podocalyxin**			
Yes	3 (37.5)	5 (6.3)	
No	5 (62.5)	75 (93.8)	**0.023**

*Stage IV patients were excluded from the study.

**Two patients experienced a complete response to NAT, one of whom had dysplastic changes and the tumor size was determined.

***Two patients lacked grade and resection margin information and two patients experienced a complete response, such that the grade could not be determined.

Linear-by-linear and Chi square -tests were used for this table.

### Podocalyxin expression

NAT patients exhibited a weaker (p = 0.049) and less strong podocalyxin immunoexpression compared to US patients using mAb (p = 0.017). In the combined mAb and pAb podocalyxin expression category we identified a statistical difference between the two groups so that the NAT patients had weak podocalyxin immunoexpression (p = 0.032) and US patients had moderate podocalyxin immunoexpression (p = 0.005). Using pAb staining, we detected no significant difference between the groups. Table 2 summarizes the podocalyxin expression distribution.

**Table 2:** Podocalyxin staining intensities using polyclonal (pAb), monoclonal (mAb) and combined antibodies comparing the neoadjuvant (NAT) and upfront surgery (US) groups.

n (%)	NAT	US	p value
**pAb**			
Strong	15 (17.0)	16 (11.2)	0.235
Moderate	30 (34.1)	63 (44.1)	0.167
Weak	25 (28.4)	44 (30.8)	0.768
Negative	18 (20.5)	20 (14.0)	0.206
**mAb**			
Strong	13 (14.8)	41 (28.7)	**0.017**
Moderate	35 (39.8)	49 (34.3)	0.402
Weak	32 (36.4)	36 (25.2)	**0.049**
Negative	8 (9.1)	17 (11.9)	0.663
**Combined**			
Strong	7 (8.0)	5 (3.5)	0.220
Moderate	14 (15.9)	47 (32.9)	**0.005**
Weak	67 (76.1)	91 (63.6)	**0.032**

Linear-by-linear test was used for this table.

In the NAT group, weaker podocalyxin immunoexpression using pAb was associated with R0 resection margin (p = 0.023), lower grade (p = 0.034), and less perineural invasion (p = 0.003). We also detected an association between NAT radiation therapy and weaker podocalyxin immunoexpression (p = 0.036) using mAb. We found no association between sex, age, vascular resection, stage, T-class, lymph node ratio (N), and perivascular invasion and either staining method (Table 3).

**Table 3:** Association between patient characteristics and podocalyxin using the polyclonal (pAb) and the monoclonal antibody (mAb) in the neoadjuvant group.

n (%)	Podocalyxin, pAb					Podocalyxin, mAb				
	Negative	Weak	Moderate	Strong	p value	Negative	Weak	Moderate	Strong	p value
**Sex**										
Female	8 (44.4)	10 (40.0)	23 (76.6)	8 (53.3)		4 (50.0)	17 (53.1)	15 (42.9)	5 (38.5)	
Male	10 (55.6)	15 (60.0)	7 (23.3)	7 (46.7)	0.104	4 (50.0)	15 (46.9)	20 (57.1)	8 (61.5)	0.531
**Age**										
< 65	8 (44.4)	13 (52.0)	13 (43.3)	8 (53.3)		2 (25.0)	17 (53.1)	18 (51.4)	5 (38.5)	
≥ 65	10 (55.6)	12 (48.0)	17 (56.7)	7 (46.7)	0.839	6 (75.0)	15 (46.9)	17 (48.6)	8 (61.5)	0.860
**Vascular resection**										
Yes	11 (61.1)	10 (40.0)	19 (63.3)	10 (66.7)		4 (50.0)	16 (50.0)	22 (62.9)	8 (61.5)	
No	7 (38.9)	15 (60.0)	11 (36.7)	5 (33.3)	0.376	4 (50.0)	16 (50.0)	13 (37.1)	5 (38.5)	0.326
**Radiation therapy**										
Yes	5 (27.8)	8 (32.0)	9 (30.0)	4 (26.7)		5 (62.5)	10 (31.3)	9 (25.7)	2 (15.4)	
No	13 (72.2)	17 (68.0)	21 (70.0)	11 (73.3)	0.924	3 (37.5)	22 (68.8)	26 (74.3)	11 (84.6)	**0.036**
**Stage**										
IA	5 (27.8)	4 (16.7)	0 (0.0)	1 (6.7)		3 (42.9)	3 (9.4)	4 (11.4)	0 (0.0%)	
IB	5 (27.8)	5 (20.8)	9 (30.0)	6 (40.0)		4 (57.1)	8 (25.0)	6 (17.1)	7 (53.8)	
IIA	2 (11.1)	3 (12.5)	1 (3.3)	1 (6.7)		0 (0.0)	3 (9.4)	3 (8.6)	1 (7.7)	
IIB	4 (22.2)	9 (37.5)	12 (40.0)	5 (33.3)		0 (0.0)	11 (34.4)	15 (42.9)	4 (30.8)	
III	2 (11.1)	3 (12.5)	8 (26.7)	2 (13.3)	0.096	0 (0.0)	7 (21.9)	7 (20.0)	1 (7.7)	0.186
IV*										
Missing**	1					1				
**T**										
T1	5 (27.8)	8 (32.0)	5 (16.7)	2 (13.3)		3 (37.5)	8 (25.0)	8 (22.9)	1 (7.7)	
T2	9 (50.0)	11 (44.0)	17 (56.7)	10 (66.7)		4 (50.0)	18 (56.3)	17 (48.6)	8 (61.5)	
T3	4 (22.2)	4 (16.0)	6 (20.0)	3 (20.0)		0 (0.0)	5 (15.6)	8 (22.9)	4 (30.8)	
T4	0 (0.0)	1 (4.0)	2 (6.7)	0 (0.0)	0.703	0 (0.0)	1 (3.1)	2 (5.7)	0 (0.0)	0.313
Missing**	1					1				
**N**										
N0	12 (66.7)	13 (52.0)	11 (36.7)	8 (53.3)		7 (87.5)	14 (43.8)	15 (42.9)	8 (61.5)	
N1	4 (22.2)	10 (40.0)	13 (43.3)	5 (33.3)		1 (12.5)	12 (37.5)	15 (42.9)	4 (30.8)	
N2	2 (11.1)	2 (8.0)	6 (20.0)	2 (13.3)	0.519	0 (0.0)	6 (18.8)	5 (14.3)	1 (7.7)	0.229
**Grade**										
1	5 (27.8)	5 (20.0)	2 (6.7)	1 (6.7)		3 (37.5)	3 (9.4)	2 (15.4)	13 (14.8)	
2	8 (44.4)	17 (68.0)	21 (70.0)	9 (60.0)		2 (25.0)	22 (68.8)	7 (53.8)	55 (62.5)	
3	3 (16.7)	3 (12.0)	6 (20.0)	4 (26.7)	**0.034**	2 (25.0)	5 (15.6)	4 (30.8)	16 (18.2)	0.392
Missing***	4					4				
**Resection margin**										
R0	18 (100.0)	19 (76.0)	19 (65.5)	10 (71.4)		7 (87.5)	24 (77.4)	27 (77.1)	8 (66.7)	
R1	0 (0.0)	6 (24.0)	10 (34.5)	4 (28.6)	**0.023**	1 (12.5)	7 (22.6)	8 (22.9)	4 (33.3)	0.343
Missing****	2					2				
**Perineural invasion**										
Yes	7 (38.9)	17 (68.0)	25 (83.3)	12 (80.0)		3 (37.5)	23 (71.9)	24 (68.6)	11 (84.6)	
No	11 (61.1)	8 (32.0)	5 (16.7)	3 (20.0)	**0.003**	5 (62.5)	9 (28.1)	11 (31.4)	2 (15.4)	0.089
**Perivascular invasion**										
Yes	3 (16.7)	6 (24.0)	13 (43.3)	3 (20.0)		0 (0.0)	7 (21.9)	15 (42.9)	3 (23.1)	
No	15 (83.3)	19 (76.0)	17 (56.7)	12 (80.0)	0.339	8 (100.0)	25 (78.1)	20 (57.1)	10 (76.9)	0.099

### Podocalyxin and survival

**Figure 3:**
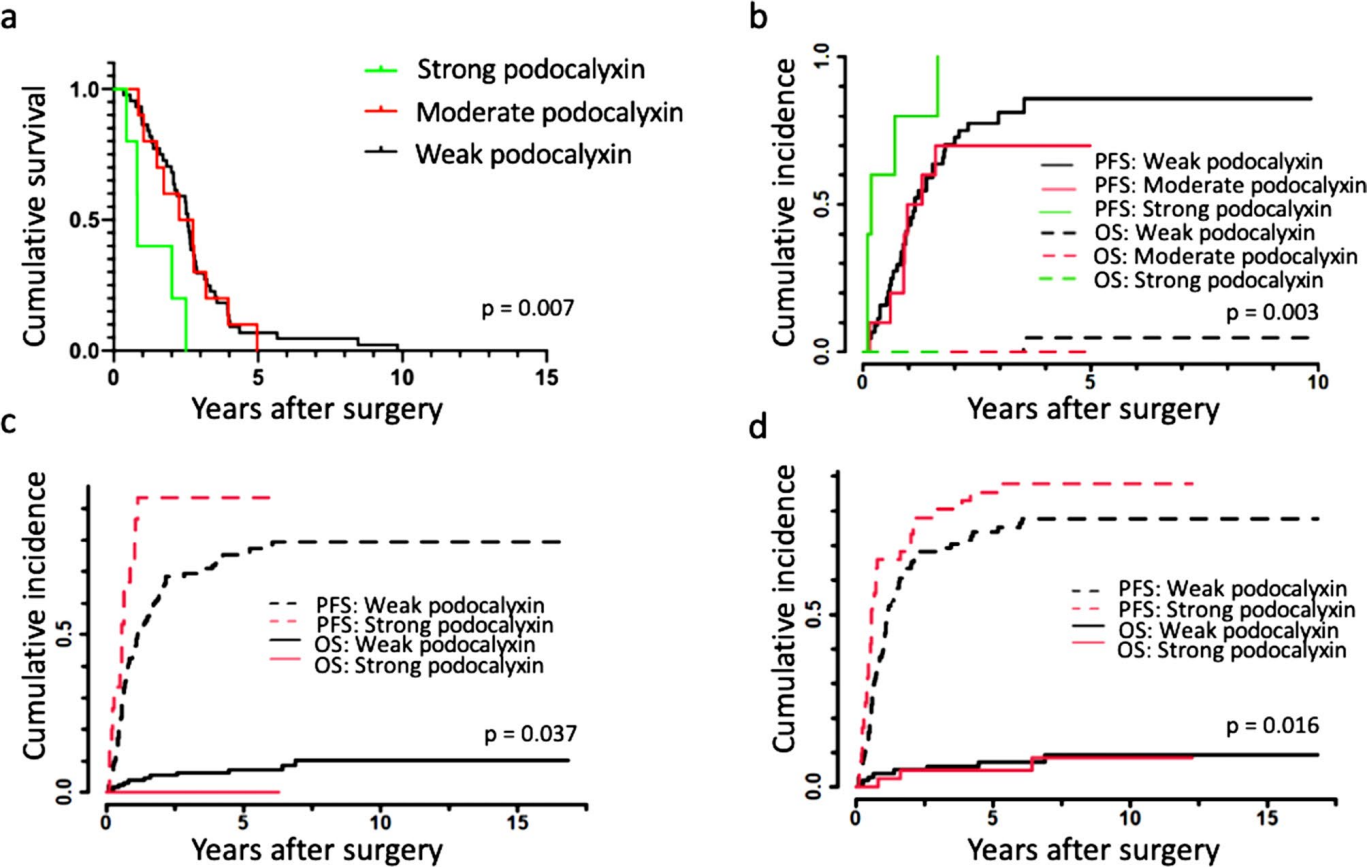
Kaplan–Meier and Aalen-Johansen survival by podocalyxin immunoexpression. (**a**) The association of disease-specific survival (DSS) and podocalyxin immunoexpression based on polyclonal (pAb), HPA2110 and monoclonal antibodies (mAb), HES9 combined among non-responders. A categorization for podocalyxin expression with three groups was created as follows: 1, strong staining for both pAb and mAb (both scored 3); 2, either exhibiting strong staining (either scored 3); and 3, both staining weakly (both scored 0–2), corresponding to strong, moderate, and weak, respectively. Non-responders were defined as a class 4 or 5 response corresponding to > 50% residual tumor cells. Kaplan–Meier analysis. Log-rank test was used for statistical significance. (**b**) The association of progression-free survival (PFS) and podocalyxin immunoexpression based on polyclonal (pAb) and monoclonal antibodies (mAb) combined among non-responders. A categorization for podocalyxin expression with three groups was created as follows: 1, strong staining for both pAb and mAb (both scored 3); 2, either exhibiting strong staining (either scored 3); and 3, both staining weakly (both scored 0–2), corresponding to strong, moderate, and weak, respectively. Non-responders were defined as a class 4 or 5 response corresponding to > 50% residual tumor cells. Death from any cause (overall survival, OS) was used as a competing event. Aalen-Johansen analysis. Gray's test was used for statistical significance. (**c**) The association of progression-free survival (PFS) and podocalyxin immunoexpression with polyclonal antibody HPA2110, (pAb) in the upfront surgery group. Podocalyxin expressions were grouped as follows: 1, strong staining for pAb (scored 3); and 2, moderate, weak or negative staining for pAb (scored 0–2) corresponding to strong and weak, respectively. Upfront surgery patients included for the analysis. Aalen-Johansen analysis. Death from any cause (overall survival, OS) was used as a competing event. Grays' test was used for statistical significance. (**d**) The association of progression-free survival (PFS) and podocalyxin immunoexpression with monoclonal antibody HES9, (mAb) in the upfront surgery group. Podocalyxin expressions were grouped as follows: 1, strong staining for mAb (scored 3); and 2, moderate, weak or negative staining for mAb (scored 0–2) corresponding to strong and weak, respectively. Upfront surgery patients included for the analysis. Aalen-Johansen analysis. Death from any cause (overall survival, OS) was used as a competing event. Grays' test was used for statistical significance.

Strong podocalyxin immunopositivity did not correlate with DSS or PFS in the entire NAT group in a univariate analysis (hazard ratio [HR] 1.45, 95% confidence interval [CI] 0.56–3.56, p = 0.466 and HR 1.19, 95% CI 0.38–3.78, p = 0.763). However, among non-responders (n = 59) (with >50% RTCs), strong podocalyxin immunopositivity associated with a poor DSS (HR 4.16, 95% CI 1.56–11.01, p = 0.004) compared to weak and moderate immunopositivity (see Fig. 3a). In addition, in the PFS univariate analysis, a strong podocalyxin immunopositivity associated with a poor outcome among non-responders (HR 3.20, 95% CI 1.02–10.07, p = 0.047), although we detected no difference between moderate and weak immunopositivity (p = 0.632; see Fig. 3b). Among responders (n = 29) (≤50% RTCs), podocalyxin did not associate with DSS or PFS.

In the DSS multivariate analysis among non-responders, a strong podocalyxin expression (HR 6.175, 95% CI 2.057–18.544, p = 0.001) associated with a poor survival. Other variables including age, stage, tumor size, adjuvant therapy, or perivascular invasion did not associate with DSS (Supplementary Table 2). In the PFS multivariate analysis of non-responders, a strong podocalyxin expression associated with a poor survival (HR 4.06, 95% CI 1.33–12.37 p = 0.014; Supplementary Table 3). We could not carry out a DSS or PFS multivariate analysis among responders given the small number of patients.

Among the US group, a strong podocalyxin immunopositivity using pAb (HR 2.31, 95% CI 1.21–4.38, p = 0.011; Fig. 3c) and mAb (HR 1.57, 95% CI 1.03–2.39, p = 0.035; Fig. 3d) associated with a poor outcome in the PFS survival analysis. In a multivariate PFS analysis, a strong podocalyxin expression (HR 7.13, 9% CI 3.30–15.44, p < 0.001), stage III (HR 1.59, 95% CI 1.01–2.51, p = 0.045), adjuvant therapy (HR 0.57, 95% CI 0.38–0.84, p = 0.005), and perivascular invasion (HR 3.10, 95% CI 2.02–4.77, p < 0.001) associated with a poor survival (Supplementary Table 4). In addition, strong podocalyxin positivity using pAb (HR 2.00, 95% CI 1.11–3.62, p = 0.022) and mAb (HR 1.77, 95% CI 1.18–2.64, p = 0.006) associated with a poor DSS. In the multivariate DSS analysis, a strong podocalyxin expression (p < 0.001), moderate podocalyxin expression (p = 0.001), perivascular invasion (p < 0.001), and adjuvant therapy (p < 0.001) associated with DSS (Supplementary Table 5).

### Response to NAT and survival

**Figure 4:**
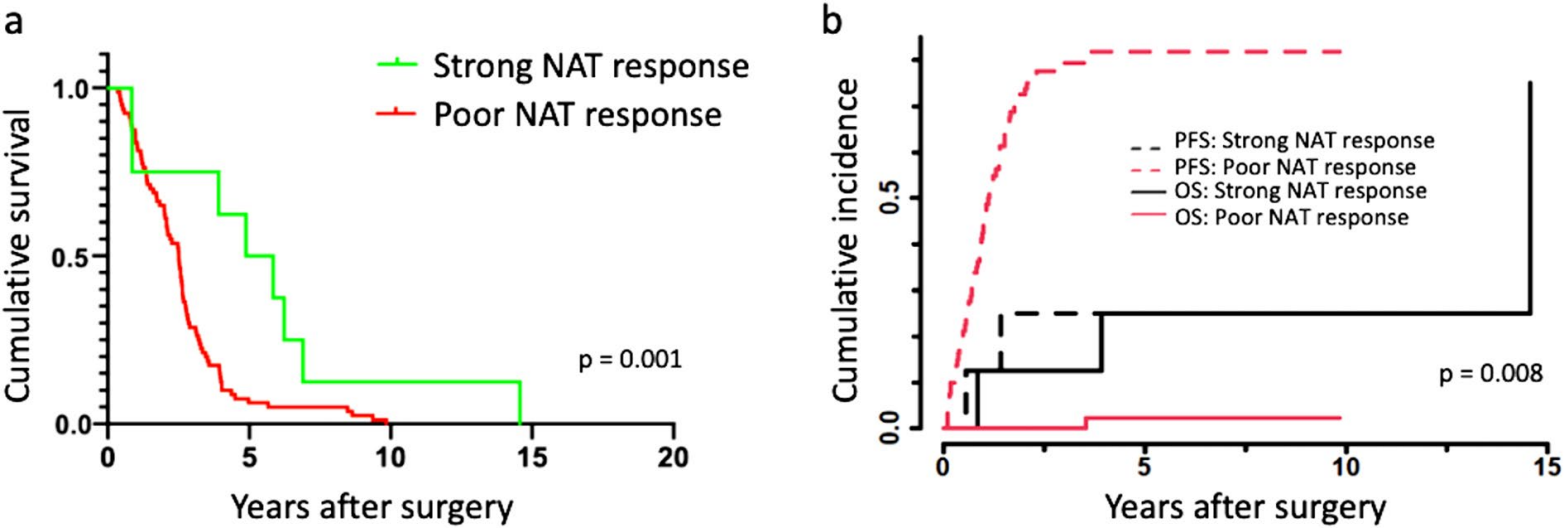
Kaplan–Meier and Aalen-Johansen survival by neoadjuvant therapy (NAT) response. (**a**) The association between NAT response and disease-specific survival (DSS). NAT responses were grouped as follows: a strong response as class 0 or 1 response, corresponding to ≤ 5% residual tumor cells (RTCs) and a poor response as class 2–5 response corresponding to > 5% RTCs. Kaplan–Meier analysis. Log-rank test was used for statistical significance. (**b**) The association between NAT response and progression-free survival (PFS). NAT responses were grouped as follows: a strong response as class 0 or 1 response, corresponding to ≤ 5% residual tumor cells (RTCs) and a poor response as class 2–5 response corresponding to > 5% RTCs Death from any cause (overall survival, OS) was used as a competing event. Aalen-Johansen analysis. Gray's test was used for statistical significance.

A strong response to NAT (≤5% RTCs) associated with a better outcome in the DSS analysis (HR 0.28, 95% CI 0.09–0.94, p = 0.039; Fig. 4a). We failed to detect a significant difference in survival between the response group with ≤10% RTCs compared to the group with >10% RTCs (HR 0.52, 95% 0.254–1.07, p = 0.074). Furthermore, we identified no difference between the groups with ≤50% and >50% RTCs (HR 1.52, 95% CI 0.86–2.70, p = 0.146) or between the groups with ≤90% and >90% RTCs (HR 1.45, 95% CI 0.87–2.40, p = 0.150).

A strong response to NAT (≤5% RTCs) associated with a better outcome in the PFS survival analysis (HR 0.18, 95% CI 0.04–0.78, p = 0.022; Fig. 4b). Interestingly, we detected a difference between the groups with ≤10% and >10% RTCs in the PFS analysis (HR 0.39, 95% 0.18–0.84, p = 0.016), but not between the groups with ≤50% and >50% RTCs (HR 1.57, 95% CI 0.90–2.75, p = 0.112) or between the groups with ≤90% and >90% of RTCs (HR 1.61, 95% CI 0.997 2.60, p = 0.051).

## Discussion

We demonstrate that podocalyxin overexpression in PDAC serves as an independent prognostic marker for poor prognosis in patients treated with NAT only if no significant response to NAT was recorded, whereby more than 50% of the viable residual tumor cells appeared in the post-pancreatectomy specimen. When the response to NAT was moderate to complete, with 50% or less viable residual tumor cells, no association between podocalyxin expression and DSS was found. In addition, podocalyxin expression associated with poor clinicopathological factors in the NAT group. In general, NAT patients exhibited a weaker podocalyxin expression compared to US patients using mAb. We also demonstrated that podocalyxin overexpression serves as an independent prognostic factor for poor progression-free survival among NAT patients only if the response to NAT was insubstantial, such that more than 50% of the viable residual tumor cells could be seen post-pancreatectomy.

Podocalyxin expression and its association with a poor survival in PDAC was described previously^26–31^. For example, Saukkonen et al. (2015) showed that strong podocalyxin expression independently served as a prognostic factor for a poor prognosis in PDAC in a study that used the same antibodies. However, patients receiving NAT were excluded and, to our knowledge, our study represents the first report describing the expression of podocalyxin in neoadjuvant-treated PDAC patients.

While podocalyxin serves as an independent marker of poor prognosis in treatment-naïve PDAC, the underlying mechanisms are not well understood. The upregulation of podocalyxin appears necessary for the epithelial–mesenchymal transition, characterized by migratory and invasive behavior and the involvement metastatic events in cancer^41–43^. Podocalyxin has been shown to promote cancer cell proliferation, prevent apoptosis, promote tumorigenesis and participate in cancer cell renewal^40,41,49,50^. Some studies suggest that metastatic, circulating PDAC cells employ podocalyxin as a functional E- and L-selectin ligand promoting cell adhesion to vascular endothelia in metastasis^27,29^. However, the causal relationship between a weaker podocalyxin expression and a better NAT response remains unclear. In PDAC, a preoperative tumor biopsy is difficult to obtain; thus, the preoperative podocalyxin expression is unknown. A comparative analysis of podocalyxin expression or the tumor cell density evaluation of the NAT response between the preoperative and postoperative tumor samples could be performed. Thus, we cannot draw a definitive conclusion regarding whether a weaker podocalyxin expression leads to a better NAT response or, alternatively, if a better NAT response leads to a weaker podocalyxin expression. Among poorly responding NAT patients, a strong podocalyxin expression associated with poor survival, similar to observations for treatment-naïve PDAC. Furthermore, in these patients, podocalyxin could be used as a survival marker. Among strong NAT responders, podocalyxin expression becomes generally weaker. Hence, podocalyxin can be used as a response marker, but is of no use as a survival marker in strong responders.

Furthermore, we show that podocalyxin serves as an independent prognostic factor of poor progression-free survival in US patients. This supports findings from an earlier study of podocalyxin and DSS among US patients^28^. Here, we detected a difference in survival, both DSS and PFS, between strong and weak podocalyxin expression, but not between moderate and weak podocalyxin expression, and these results agree with previous findings. Saukkonen et al. showed that strong podocalyxin expression compared to weak podocalyxin expression was associated with poor survival^28^.

In addition, we found that only 2 (2.3%) patients exhibited a complete response to NAT. Chatterjee at al. described a similar complete response rate of 2.7%^16^ while White at al. described a complete response rate of 6%^14^. We detected a significant association between the NAT response and survival between groups with 5% or fewer and groups with more than 5% residual tumor cells. We found no difference, however, in survival between groups with ≤10% and >10% RTCs, the groups with ≤50% and >50% RTCs, or the groups with ≤90% and >90% of RTCs. Thus, when using NAT, improvement in survival may only occur if the response to NAT is nearly complete, such that no more than 5% of residual tumor cells appear in the surgical specimen. A strong response to NAT was also associated with a better stage, T-class, lymph node ratio, grade, perineural invasion, and negative podocalyxin expression. Patients treated with NAT radiotherapy significantly more often exhibited a strong response to NAT, supporting its use as a part of NAT. In the study of Chatterjee et al. patients treated with neoadjuvant chemotherapy instead of chemoradiation had more residual tumor cells in the post-pancreatectomy specimen^16^.

We designed a six-tier scheme to grade the response to NAT based on the percentage of viable residual tumor cells observed in the post-pancreatectomy specimen. This scheme mirrors previously established schemes. For example, Ishikawa et al. (1989) proposed a grading system with three categories based on the percentage of severely degenerative cancer cells: less than one-third, one-third to two-thirds, and more than two-thirds^11^. Evans et al. (1992) proposed a four-tiered system based on assessing the percentage of tumor cell destruction: I, destruction of <10% tumor cells; IIa, destruction of 10% to 50% of tumor cells; IIb, destruction of 51% to 90% of tumor cells; III, <10% viable tumor cells (>90% destroyed); and IV, no viable tumor cells^12^. White et al. (2005) proposed a three-tier system similar to Evans based on the percentage of the remaining viable tumor cells: <10, 10% to 90%, and >90%^13^. The College of American Pathologists (CAP) proposes using four categories: 0, no viable tumor cells; 1, single cells or small groups of cancer cells; 2, residual cancer outgrown by fibrosis; and 3, minimal or no tumor kill^14^. The CAP and Evans grading schemes appear to correlate with overall and DFS^15,17^.

Chatterjee et al. (2012) used the Evans and CAP grading systems, identifying a difference in survival between patients with only minimal residual tumor cells compared to patients with a moderate to poor response^16^. They found no difference between Evans grade I, IIa, and IIb or CAP grades 2 and 3. Thus, a three-tier grading system has been proposed: no residual carcinoma, <5% residual carcinoma, and >5% residual carcinoma^16^. By contrast, in the study by White et al. (2005), patients with a large residual tumor (class 5) experienced a shorter survival time than those with a moderate or good response to NAT^13^.

In our progression-free survival analysis, we detected a difference between groups with 5% or less and more than 5% residual tumor cells, observing an additional difference between groups with 10% or less and more than 10% residual tumor cells, but not between the other groups. Chatterjee et al. recommended using a three-tier grading system to group responses to complete, <5%, and >5% residual tumor cells. Our results regarding the progression-free survival identified a difference between groups with <10% and >10% residual tumor cells, suggesting that perhaps additional groups are warranted.

One strength to our study lies in its relatively large, well-described cohort of patients with histologically determined PDAC. We excluded other tumors of the pancreas or cancers originating from the bile ducts. The NAT and US groups were matched for age, sex, and time of surgery. Moreover, our data on the survival and follow-up are reliable and precise. To evaluate the neoadjuvant response, we designed the six-tier scheme adapting it from previously designed schemes. Our scheme follows the recommendations of the latest tumor response consensus statements from the Amsterdam International Consensus Meeting^19^. Furthermore, multiple representative samples for each patient were reviewed to evaluate the NAT response. Given these strengths, we acknowledge limitations to our study. The TMA samples are relatively small (1-mm diameter) and, thus, the representativeness of the cancer and podocalyxin staining may have suffered. Specifically, podocalyxin may be unevenly distributed in the tumor. These limitations were minimized by taking several (6) TMA samples per tumor and by scoring the podocalyxin intensity using the strongest intensity visible in the tumor. An experienced pathologist determined the TMA drilling spots on the specimen. Furthermore, the podocalyxin intensity scoring was determined by two independent investigators in order to minimize the effect of subjectivity. This study was conducted to clarify the relationship between podocalyxin staining and NAT, considering the response to NAT. Due to the retrospective nature of our study, implementation of NAT varies between patients. This study featured a limited number of patients with a good or strong response to NAT and, thus, we could not perform a multivariate survival analysis for those patients.

## Conclusions

To our knowledge, this is the first report to show that podocalyxin serves as an independent predictor of a poor prognosis in neoadjuvant-treated PDAC patients, but only if the response to NAT is not significant. The causal relationship between a better NAT response, decreased podocalyxin expression, and an inability of podocalyxin to serve as a prognostic marker in NAT patients who respond well remains unclear. A complete or nearly complete response to NAT is rare, whereby those patients seem to benefit from NAT in terms of survival.

## Supplementary Information

Supplementary Information.

## Data Availability

Data supporting the results of this study is available upon request to the corresponding author.
