# Supplementary material for: Impact of histological response after neoadjuvant therapy on podocalyxin as a prognostic marker in pancreatic cancer
Source: Sci Rep. 2021 May 10;11:9896. doi: 10.1038/s41598-021-89134-2 (PMC8110523; doi:10.1038/s41598-021-89134-2)
Supplement: Supplementary file 1 — Supplementary Information. [file 41598_2021_89134_MOESM1_ESM.pdf]

Supplementary Data

Impact of histological response after neoadjuvant therapy on podocalyxin as a prognostic marker in pancreatic cancer

Annika Eurola[1], Ari Ristimäki[2,3], Harri Mustonen[1], Anna-Maria Nurmi[1], Jaana Hagström[2,4], Caj Haglund*[1] and Hanna Seppänen*[1]

[1]Department of Surgery, Translational Cancer Medicine Research Program, Faculty of Medicine, University of Helsinki and Helsinki University Hospital, Helsinki, Finland
[2]Department of Pathology, HUSLAB, HUS Diagnostic Center, University of Helsinki and Helsinki University Hospital, Helsinki, Finland
[3]Applied Tumor Genomics (ATG), Research Programs Unit, Faculty of Medicine, University of Helsinki, Helsinki, Finland
[4]Department of Oral Pathology and Radiology, University of Turku, Turku, Finland
*Shared equal last authorship

Supplementary Table 1.

Neoadjuvant treatments used.

	n (%)
Cisplatin–gemcitabine* (with or without radiation)	43 (48.9%)
Single gemcitabine (with or without radiation)	25 (28.4%)
FOLFIRINOX** (with or without radiation)	13 (14.8%)
Nab-paclitaxel–gemcitabine	5 (5.7%)
Capecitabine–gemcitabine	1 (1.1%)
Only radiation	1 (1.1%)
Radiation therapy used	26 (29.5%)
**N (total)**	**88 (100%)**

*One patient received Capecitabine after Cisplatin–Gemcitabine
**One patient received Cisplatin–Gemcitabine + radiation and one patient received Nab-paclitaxel–gemcitabine after FOLFIRINOX.

Supplementary Table 2.

Disease spesific survival multivariate analysis among non-responders.

	HR	95% CI	p value
**Age**	1,008	0,964–1,053	0,735
**Stage III**	1,639	0,751–3,579	0,215
**Tumor size**	1,005	0,979–1,033	0,693
**Adjuvant therapy**	0,559	0,271–1,151	0,114
**Perivascular invasion**	1,661	0,751–3,674	0,211
**Podocalyxin**			
weak	1		
moderate	1,293	0,528–3,167	0,574
strong	6,175	2,057–18,544	**0,001**

Abbreviations HR = Hazard ratio, CI = Confidence interval

Supplementary Table 3.

Disease free survival multivariate analysis among non-responders.

	HR	95% CI	p value
**Age**	1,008	0,963–1,055	0,737
**Stage III**	1,963	0,824–4,674	0,128
**Tumor size**	1,008	0,977–1,040	0,614
**Adjuvant**	0,600	0,300–1,197	0,147
**Perivascular invasion**	1,127	0,513–2,478	0,765
**Podocalyxin**			
weak	1		
moderate	0,933	0,397–2,193	0,874
strong	4,057	1,330–12,375	**0,014**

Abbreviations HR = Hazard ratio, CI = Confidence interval

Supplementary Table 4.

Progression free survival multivariate analysis among upfront surgery patients.

	HR	95% CI	p value
**Age**	1,001	0,980–1,024	0,897
**Stage III**	1,595	1,011–2,516	**0,045**
**Tumor size**	1,006	0,991–1,020	0,448
**Adjuvant**	0,568	0,382–0,846	0,005
**Perivascular invasion**	3,105	2,019–4,775	**<0.001**
**Podocalyxin**			
weak	1		
moderate	1,603	1,065–2,413	**0,024**
strong	7,135	3,297–15,444	**<0.001**

Abbreviations HR = Hazard ratio, CI = Confidence interval

Supplementary Table 5.

Disease spesific multivariate analysis among upfront surgery patients.

	HR	95% CI	p value
**Age**	1,013	0,989–1,038	0,291
**Stage III**	1,482	0,975–2,253	0,066
**Tumor size**	1,013	0,998–1,027	0,085
**Adjuvant**	0,455	0,304–0,681	**<0.001**
**Perivascular invasion**	2,537	1,730–3,722	**<0.001**
**Podocalyxin**			
weak	1		
moderate	2,041	1,364–3,056	**0,001**
strong	4,165	2,601–6,669	**<0.001**

Abbreviations HR = Hazard ratio, CI = Confidence interval
